# Unwinding of synthetic replication and recombination substrates by Srs2

**DOI:** 10.1016/j.dnarep.2012.05.007

**Published:** 2012-10-01

**Authors:** Victoria Marini, Lumir Krejci

**Affiliations:** a Department of Biology, Masaryk University, Kamenice 5/A7, 625 00 Brno, Czech Republic; b National Centre for Biomolecular Research, Masaryk University, Kamenice 5/A4, 625 00 Brno, Czech Republic; c International Clinical Research Center, Center for Biomolecular and Cellular Engineering, St. Anne's University Hospital Brno, Brno, Czech Republic

**Keywords:** DNA repair, Recombination, Srs2, Replication, Helicase

## Abstract

The budding yeast Srs2 protein possesses 3′ to 5′ DNA helicase activity and channels untimely recombination to post-replication repair by removing Rad51 from ssDNA. However, it also promotes recombination via a synthesis-dependent strand-annealing pathway (SDSA). Furthermore, at the replication fork, Srs2 is required for fork progression and prevents the instability of trinucleotide repeats. To better understand the multiple roles of the Srs2 helicase during these processes, we analysed the ability of Srs2 to bind and unwind various DNA substrates that mimic structures present during DNA replication and recombination. While leading or lagging strands were efficiently unwound, the presence of ssDNA binding protein RPA presented an obstacle for Srs2 translocation. We also tested the preferred directionality of unwinding of various substrates and studied the effect of Rad51 and Mre11 proteins on Srs2 helicase activity. These biochemical results help us understand the possible role of Srs2 in the processing of stalled or blocked replication forks as a part of post-replication repair as well as homologous recombination (HR).

## Highlights

► Srs2 shows binding preference for ssDNA–dsDNA junction. ► Srs2 unwinds branch-containing structures. ► RPA inhibits the unwinding of Srs2. ► Srs2 unwinds recombination intermediates.

## 1 Introduction

The process of homologous recombination (HR) is widespread in nature, as it is present in organisms from bacteria to humans. It contributes to genetic diversity and is also essential for maintaining the integrity of the genome through facilitating the repair of DNA double-strand breaks (DSBs) and restarting stalled replication forks. DSBs are caused by a vast number of endogenous and exogenous agents, including genotoxic chemicals and ionising radiation or the replication of a damaged template [1]. If not repaired, the DSBs can lead to aneuploidy, genetic aberrations or cell death [2–4].

DSB repair by HR is initiated by the resection of 5′ ends on each side of the DSB, leading to the formation of 3′ single-stranded tails covered by the single-strand binding protein RPA. Through the activity of Rad52, the RPA protein is replaced by Rad51, forming the presynaptic filament [5]. This filament, together with the help of Rad54 protein, engages in a homology search and strand invasion (D-loop formation) followed by DNA synthesis [5]. At this point, the recombination repair mechanism can proceed via two alternative subpathways. The first, known as the general DSB repair pathway (DSBR), is characterised by the capture of the second 3′ tail and a consequent new round of DNA synthesis and ligation, resulting in the formation of a double Holliday junction (dHJ). This structure can then be either dissolved by the action of the Sgs1/Top3 complex to produce noncrossover products or resolved to produce either noncrossover or crossover products [1]. In the alternative pathway (SDSA), the second 3′ tail is annealed to the invading strand as it is released from the D-loop structure after its extension by DNA synthesis [1,6].

Several helicases have been implicated in the repair of stalled replication forks [1,7]. Of these helicases, Srs2 seems to play several important and sometimes opposing roles [7,8]. The *SRS2* gene of *Saccharomyces cerevisiae* encodes a DNA helicase belonging to superfamily I and shows homology to the bacterial helicases Rep, PcrA and UvrD [9–11]. While the homologous helicase domain is located at the N-terminus, the C-terminal third of the protein bears no homology to the *Escherichia coli* Srs2 homologues and has been implicated in several protein interactions and regulation of multiple processes [7]. The Srs2 protein has both ssDNA-dependent ATPase, DNA helicase activities in the 3′-5′ direction, and is able to unwind various DNA substrates [12–14]. The *SRS2* gene was first identified as a suppressor of radiation-sensitive mutations, i.e., the *rad6* and *rad18* mutants [9,15], and also as hyperecombination gene 5 [16,17]. Moreover, the deletion of *SRS2* confers lethality or strong growth defects when combined with the deletion of several genes involved in DNA repair [7]. This synthetic lethality as well as the suppression of the UV sensitivity of post replication repair (PRR) mutants and the hyperecombination phenotype are all alleviated by the deletion of genes from the *RAD52* epistasis group [16,18–21]. This suggests that Srs2 has a role in preventing undesirable homologous recombination by channelling repair into a non-recombinogenic pathway [18]. The anti-recombinase function of Srs2 was confirmed in vitro by direct inhibition of Rad51-mediated reactions including strand exchange and D-loop [22,23]. Additional experiments then demonstrated ability of Srs2 to dismantle the Rad51 presynaptic filament [22,23] and influence the formation of Rad51 foci in vivo [24]. This anti-recombination activity requires translocase activity that is fully dependent on ATP hydrolysis [25]. Moreover, it was found that Srs2 stimulates ATP hydrolysis within the Rad51 filament through a physical interaction with Rad51, which leads to the release of Rad51 from DNA [26]. In addition, an Srs2 mutant that has lost its ability to interact with the Rad51 protein is unable to disrupt the Rad51 nucleoprotein filament [26,27]. In turn, Rad51 was shown to stimulate Srs2 helicase activity [14]. Srs2 was also shown to antagonise the ability of Rad52 to mediate Rad51 filament formation by directly competing for the overlapping interaction site [28].

Srs2 was also shown to be necessary for the efficient repair of double-strand breaks through homologous recombination by promoting the SDSA pathway, thus preventing the formation of crossover products during mitosis [2,29]. The phosphorylation of Srs2 was recently suggested to play an important role in this process [30]. One scenario suggested that Srs2 can directly dissolve the D-loop structure; however, Mph1 has been shown to be more adept at doing so [14,31,32]. In an alternative scenario, Srs2 might prevent second end capture or collaborate with nucleases to cleave D-loops, DNA tails or other intermediates after re-annealing a displaced extended strand. Furthermore, Srs2 localises not only to repair centres but also to replication forks [24,33]. This recruitment relies on an interaction with sumoylated PCNA through SUMO-and PCNA-interaction motifs [24,33–36]. However, the role of Srs2 at the replication fork does not seem to be the prevention of HR only. Additionally, the replication checkpoint was shown to inhibit the recruitment of the Rad52 mediator, which is responsible for loading the Rad51 recombinase. The Rad51 foci that are formed in the absence of Rad52 do not represent functional recombination filaments [24]. These data indicate an additional role for Srs2 at the replication fork. Indeed, the overproduction of either Srs2 or its helicase-dead mutant results in a synthetic lethal phenotype in combination with many replication-associated genes [37]. Furthermore, Srs2 was found to be important for the prevention of fragility and instability of trinucleotide repeats, most likely by facilitating replication through the hairpin structures, as it was shown to preferentially unwind CTG and CGG hairpins [38–41]. To better understand the multiple roles of the Srs2 helicase during these processes, we analysed the ability of Srs2 to bind and unwind various DNA substrates that mimic the structures present during DNA replication and recombination. In addition, we tested the preferred directionality of the unwinding of various replication fork-mimicking substrates and studied the effect of the RPA, Rad51, and Mre11 proteins on Srs2 helicase activity.

## 2 Materials and methods

### 2.1 Purification of wt and ΔC forms of Srs2

For expression of wt Srs2 and Srs2ΔC, corresponding to the fragment 1-898 and containing a His_9_ affinity tag [26,33], BL21 cells carrying the plasmids (pET11d) were grown in 2xTY media containing 0.1 mg/ml ampicillin at 37 °C to an *A*_600_ of 1. The temperature was shifted to 16 °C and the cultures were induced with 0.1 mM isopropyl-1-thio-β-d-galactopyranoside overnight. Purification was performed as described previously [27]. Cells were harvested by centrifugation and the pellet resuspended in lysis buffer C (50 mM Tris pH 7.5, 10% sucrose, 10 mM EDTA, 1 mM DTT, 0.01% NP40, protease inhibitors: 2 μg/ml aprotinin, 5 μg/ml benzamidine hydrochloride, 10 μM chymostatin, 10 μM leupeptin hemisulphate, 1 μM pepstatin A) containing 600 mM KCl and lysed by sonication. All consequent purification steps were performed at 4 °C. The clarified lysate was subjected to precipitation with ammonium sulphate (208 mg/ml). The precipitate was diluted with buffer K (20 mM K_2_HPO_4_ pH 7.5, 10% glycerol, 0.5 M EDTA) to a conductivity corresponding to buffer K containing 150 mM KCl. The solution was then loaded onto a 7 ml Q Sepharose column and the resulting flow was immediately loaded onto a 7 ml SP sepharose column (GE Healthcare Life Sciences). The SP column was developed with a 70 ml gradient of 150–1000 mM KCl. The fractions containing Srs2 were mixed with Ni-NTA agarose (Qiagen) overnight. The proteins were eluted in a step-gradient using 50, 150, 300 and 500 mM imidazole in buffer K. Srs2 was mainly eluted at 150–300 mM imidazole. The fractions containing Srs2 were loaded on a 1 ml hydroxyapatite column (BioRad). Elution was done with a 10 ml linear gradient from 50 to 700 mM KH_2_PO_4_ in buffer K. Srs2 eluted at about 350 mM KH_2_PO_4_. Peak fractions were pooled, loaded onto a 1 ml Mono S column (GE Healthcare Life Sciences), and eluted with a 10 ml gradient from 75 to 1000 mM KCl in buffer K. Individual fractions were concentrated on Centricon 30,000 MWCO (Millipore) and the protein stored at −80 °C.

### 2.2 Synthetic DNA substrates

Synthetic oligonucleotides were purchased from VBC Biotech (Austria). The sequences and the structures are shown in Table 1. Some of oligonucleotides were end modified by a fluorescent dye (fluorescein or CY5). All substrates were prepared as described in Matulova et al. [42]. In brief, the equimolar amounts of the corresponding oligonucleotides were mixed in hybridizing buffer (50 mM Tris pH 7.5, 100 mM NaCl, 10 mM MgCl_2_), heated at 85 °C for 3 min and cooled slowly to room temperature to anneal. The substrates were then purified by HPLC using a 1 ml Mono Q column (GE Healthcare Life Sciences) and eluted in 20 ml gradient of 10 mM Tris buffer containing up to 1 M NaCl. Purity of each substrate was also checked on non-denaturing PAGE. Fractions were then concentrated on Vivaspin Concentrator 5000 MWCO and washed with buffer W (25 mM Tris pH 7.5, 3 mM MgCl_2_). Concentrations were determined using absorbance at 260 nm and corresponding molar extinction coefficients, taking into account the extinction coefficients of the used fluorophores (*ɛ*_fluorescein,260_ = 13,700 l/mol cm, *ɛ*_Cy5,260_ = 10,000 l/mol cm).

### 2.3 DNA binding assay

Fluorescently labelled DNA substrate (3 nM) was incubated at 30 °C with the indicated quantities of Srs2 in buffer E (30 mM Tris pH 7.5, 1 mM DTT, 0.1 mg/ml BSA and 100 mM KCl) for 10 min. The reaction mixtures were put on ice followed by addition of loading buffer (60% glycerol, 10 mM Tris pH 7.5, 60 mM EDTA, added in a sample:buffer ratio of 5:1) and the samples were resolved by electrophoresis on a 7% native PAGE in 0.5 × TBE buffer. Gels were scanned using FLA-9000 Starion (Fujifilm) and quantified by MultiGauge software (Fujifilm).

### 2.4 Helicase assays

Fluorescently labelled DNA substrate (3 nM) was incubated at 30 °C with the indicated quantities of Srs2 in buffer H (30 mM Tris pH 7.5, 1 mM DTT, 0.1 mg/ml BSA, 100 mM KCl, 20 mM creatine phosphate, 20 μg/ml creatine kinase, 2.4 mM MgCl_2_, and 2 mM ATP) for 10 min. The reaction was stopped by addition of 2% SDS and 0.5 mg/ml proteinase K and incubated at 37 °C for 3 min. After adding loading buffer to the samples the reaction products were resolved by electrophoresis on a 12% native PAGE in TBE buffer. Gels were scanned using FLA-9000 Starion (Fujifilm) and quantified by MultiGauge software (Fujifilm). For the assays in the presence of RPA, the DNA substrate was preincubated with 190 nM of RPA for 2 min at 25 °C. For testing the effect of Rad51, the DNA substrate was preincubated with Rad51 (30, 50, 100, and 200 nM) for 5 min at 30 °C. In the case of the assay in the presence of Mre11, the DNA substrate was preincubated with Mre11 (10, 25, 50, and 250 nM) for 5 min on ice, before starting the reaction by the addition of wtSrs2.

### 2.5 Branch migration assays

Fluorescently labelled DNA substrate (6 nM) was incubated at 30 °C with the indicated quantities of Srs2ΔC, Rad5 or Mph1 in buffer D (25 mM Tris pH 7.5, 1 mM DTT, 0.1 mg/ml BSA, 50 mM KCl, 7.5 mM creatine phosphate, 11.25 μg/ml creatine kinase, 2.5 mM MgCl_2_ and 2.5 mM ATP) for 15 min. The reaction was stopped by an addition of SDS to 0.2% and proteinase K to 0.5 mg/ml followed by incubation at 30 °C for 3 min. For the reactions in the presence of RPA, DNA was incubated with RPA for 5 min on ice before addition of the helicase. After the addition of loading buffer, the reaction products were resolved by electrophoresis on a 12% native PAGE in TBE buffer. Gels were scanned using the image scanner FLA-9000 Starion (Fujifilm) and quantified by MultiGauge software (Fujifilm).

### 2.6 ATPase assay

Srs2ΔC (100 nM) was incubated with ssDNA (15 μM nucleotides), 1 mM ATP and 4 nCi/μL of [γ-^32^P] ATP at 30 °C in buffer AA (30 mM Tris pH 7.5, 0.5 mM DTT, 0.1 mg/ml BSA, 0.9 mM MgCl_2_). To assess the effect of Rad51 or RPA on the hydrolysis of ATP by Srs2ΔC, ssDNA (15 μM nucleotides) was mixed with Rad51 (650 nM) or RPA (260, 500 and 760 nM) for 5 min at 30 °C. Then, Srs2ΔC (100 nM) was added, the reaction was started by an addition of ATP followed by incubation at 30 °C. Aliquots were withdrawn at the indicated times and the reaction was stopped by adding SDS to 1%. The reaction products were separated by thin layer chromatography on cellulose plates. These were analysed by phosphorimaging using a scanner FLA-9000 Starion (Fujifilm) and the amount of labelled phosphate released during ATP hydrolysis was quantified with MultiGauge software (Fujifilm).

### 2.7 Affinity pull-down assays

To pull-down potentially formed complexes, Srs2ΔC was captured onto Ni-NTA agarose (Qiagen), specific for the His_6_ tag on Srs2ΔC. Purified Srs2ΔC (6 μg) was incubated with RPA (6 μg) in 30 μl of buffer T (20 mM Tris–HCl, pH 7.5, 100 mM KCl, 1 mM DTT, 0.5 mM EDTA, and 0.01% NP40) for 20 min at RT. The reactions were mixed with 15 μl Ni-NTA agarose and again incubated for 20 min at RT. After washing the beads with 100 μl of buffer T, the bound proteins were eluted with 30 μl of 5% SDS. As a control, RPA was also incubated only with the Ni-NTA beads, without Srs2ΔC. For the reactions in the presence of DNA, ssDNA (1 μg) and RPA were preincubated for 5 min at 30 °C previous to the addition of Srs2ΔC. After the incubation with the Ni-NTA agarose the samples were treated with DNase I (2 units, New England Biolabs) for 10 min at 37 °C. The input (I), supernatant (S), and SDS eluate (E), (5 μL each), were subject to SDS-PAGE analysis.

## 3 Results

### 3.1 Srs2ΔC shows binding preference for ssDNA–dsDNA junction

**Fig. 1:**
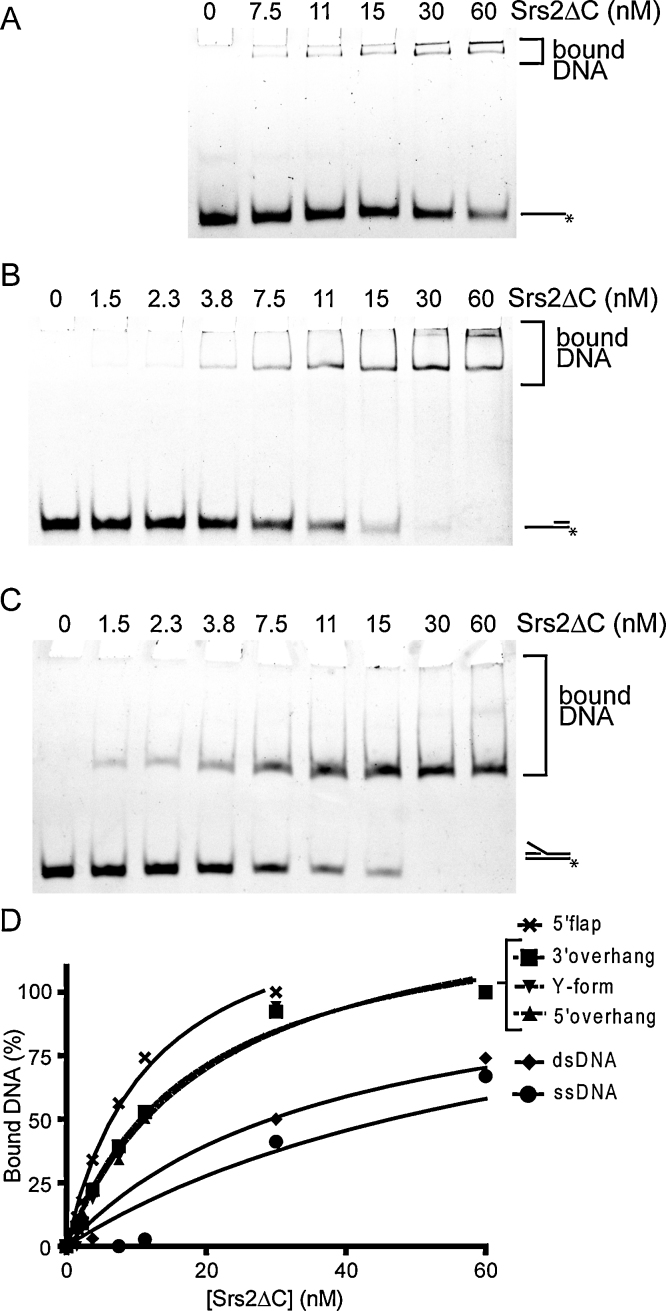
The affinity of Srs2ΔC towards a variety of DNA substrates. The binding preference of Srs2ΔC to different fluorescently labelled DNA substrates (3 nM) was studied by an electrophoretic mobility shift assay (EMSA). Reaction mixtures containing the indicated amounts of Srs2ΔC were incubated for 10 min at 30 °C. Gels represent some of the tested DNA substrates including the 3′ overhang (A), Y-form (B) and 5′ flap (C). (D) A plot of the percentage of DNA bound by Srs2ΔC, representing an average of three independent experiments.

To better understand the possible role of Srs2 during PRR and HR, we examined its binding affinity for various DNA structures that may imitate those found during DNA metabolic processes. To obtain large quantities of Srs2 and avoid a problem with aggregation, we used Srs2ΔC corresponding to the fragment 1-898, which is truncated in the non-conserved C-terminus of the protein [26]. We tested a wide range of fluorescently labelled DNA substrates, including ssDNA, blunt dsDNA, partial duplexes (3′ overhang and 5′ overhang), a fork containing the lagging (3′ flap) and the leading strand (5′ flap), both strands (fork) or the Y-form (Table 1). The DNA substrates were incubated with increasing amounts of Srs2ΔC, and the reaction mixtures were analysed by electrophoretic mobility shift assay (EMSA). As summarised in Fig. 1, we observed a comparable affinity for both dsDNA and ssDNA. However, a higher preference was detected for substrates containing an ssDNA–dsDNA junction. In particular, the 3′ overhang, 5′ overhang, and 5′ flap were bound with a much higher affinity than the other substrates tested (Fig. 1D).

**Fig. 2:**
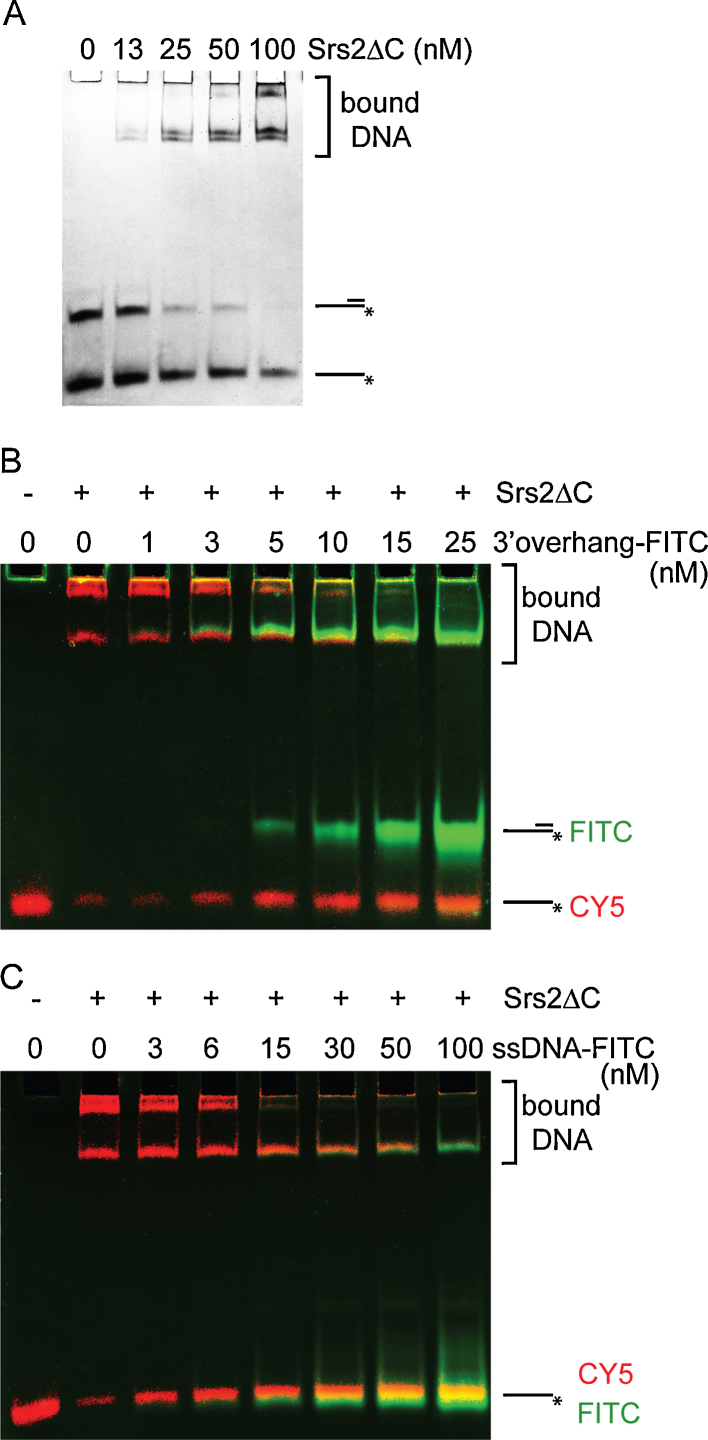
DNA binding preference using competition assay. (A) Reaction mixtures containing FITC-labelled ssDNA (3 nM) and 3′ overhang (3 nM) were incubated together with the indicated amounts of Srs2ΔC for 10 min at 30 °C and analysed by EMSA. Alternatively, the reaction mixtures containing CY5-labelled ssDNA and 150 nM Srs2ΔC were challenged with increasing amounts of FITC-3′ overhang (B) or FITC-ssDNA (C) followed by incubation for 10 min at 30 °C and then analysed. CY5-labelled substrate is shown in red and FITC-labelled substrates in green.

To validate this substrate preference, we also used two types of competition assays. First, the binding preference was analysed in reactions containing a mixture of two different DNA substrates incubated with an increasing concentration of Srs2ΔC. As shown in Fig. 2A, when mixing 3′ overhang and ssDNA, we observed a clear binding preference for the 3′ overhang. At 50 nM Srs2ΔC, almost all of 3′ overhang was in the protein-DNA complex, in contrast to the ssDNA, which remained mostly unbound at the same concentration of the protein. Second, we performed an assay where we challenged the Srs2ΔC/CY5-labelled ssDNA complex with increasing amounts of the FITC-labelled 3′ overhang competitor. We observed the release of 50% of the CY5-ssDNA from the Srs2ΔC complex at only 5 nM of the competitor (Fig. 2B). However, when the Srs2ΔC/CY5-labelled ssDNA complex was challenged with the FITC-labelled ssDNA, a 50% reduction of DNA binding was observed at a concentration of the competitor 3 times higher compared to that observed with the 3′ overhang (Fig. 2C). Taken together, these data show that Srs2ΔC demonstrates preferential binding towards DNA substrates containing an ssDNA–dsDNA junction.

### 3.2 Srs2 unwinds branch-containing structures

**Supplementary Fig. 1:**
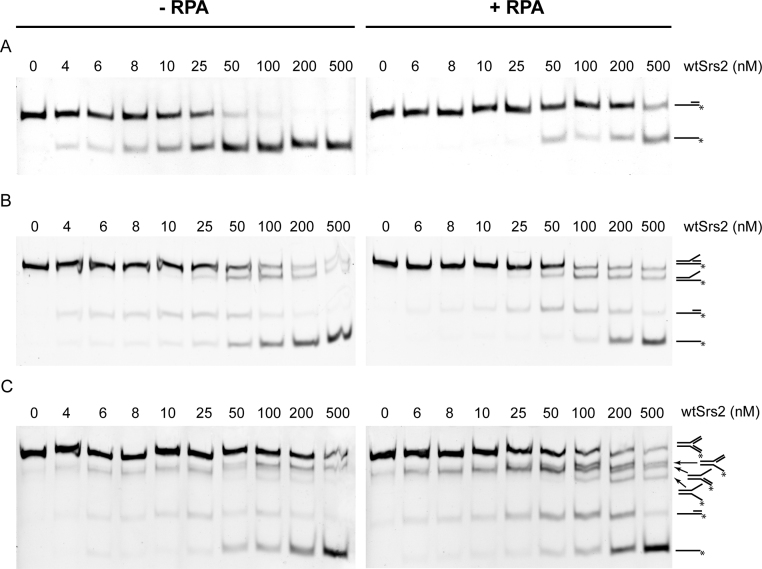
Helicase activity performed with full-length Srs2. The DNA substrate, 3′ overhang (A), 3′ flap (B) or fork (C), was first preincubated for 2 min at 25 °C either in the absence (left) or presence (right) of RPA (190 nM). Then, the indicated amount of Srs2 was added and samples were incubated for 10 min at 30 °C.

**Fig. 3:**
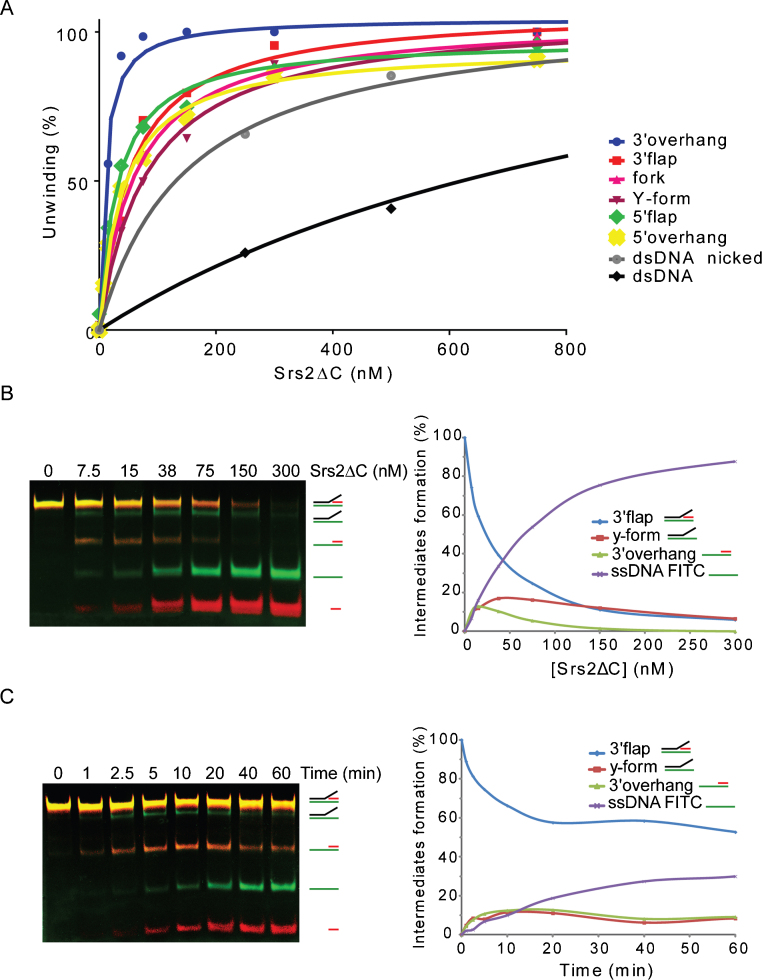
Srs2 is able to unwind more strands from fork-like structures. (A) A plot of the unwinding of the different substrates relative to the increasing concentration of Srs2ΔC. The data represent the average from at least three experiments, and curves were obtained from nonlinear regression using GraphPad Prism software. (B) The ability of Srs2ΔC to preferentially unwind strands from fork-like structures was tested by mixing 3′ flap (3 nM) labelled with both CY5 (in red) and FITC (in green) in buffer H with the indicated amounts of Srs2 and incubating for 10 min at 30 °C. The reactions were resolved using native polyacrylamide electrophoresis and analysed. The plot indicates the ratio of the different DNA intermediates (containing FITC) as a function of increasing Srs2 concentration. (C) The time-dependence of the helicase activity in which 15 nM Srs2ΔC was incubated with 3 nM 3′ flap labelled with CY5 (in red) and FITC (in green) in buffer H at 30 °C. At the indicated times, the individual aliquots were withdrawn and analysed. The plot shows the ratio of the different DNA intermediates (containing FITC dye) over time.

Next, we wished to determine whether the ability of Srs2 to unwind various DNA substrates (dsDNA, nicked dsDNA, 3′ overhang, 5′ overhang, 3′ flap, fork, Y-form, and Holliday junctions) correlates with the observed DNA binding affinities. Fluorescently labelled DNA substrates were incubated with increasing amounts of Srs2ΔC (Fig. 3A) or full-length Srs2 (Supplementary Fig. 1) followed by the resolution of the products by native PAGE. As expected, the highest efficiency of unwinding among all of the substrates was observed for the 3′ overhang (Fig. 3A). The second group of substrates with comparable unwinding included the fork, 3′ flap, Y-form, and 5′ overhang. The blunt end duplex was also unwound, but only at much higher concentrations of Srs2ΔC (Fig. 3A). Interestingly, the introduction of a nick in the dsDNA substrate resulted in a significant increase in the unwinding, almost reaching the level of other ss/dsDNA junction-containing structures (Fig. 3A). These results indicate that Srs2 is able to unwind a broad range of branch-containing DNA substrates and is most efficient with a 3′ overhang substrate.

### 3.3 Unwinding of forks with lagging and/or leading strand by Srs2ΔC

**Supplementary Fig. 2:**
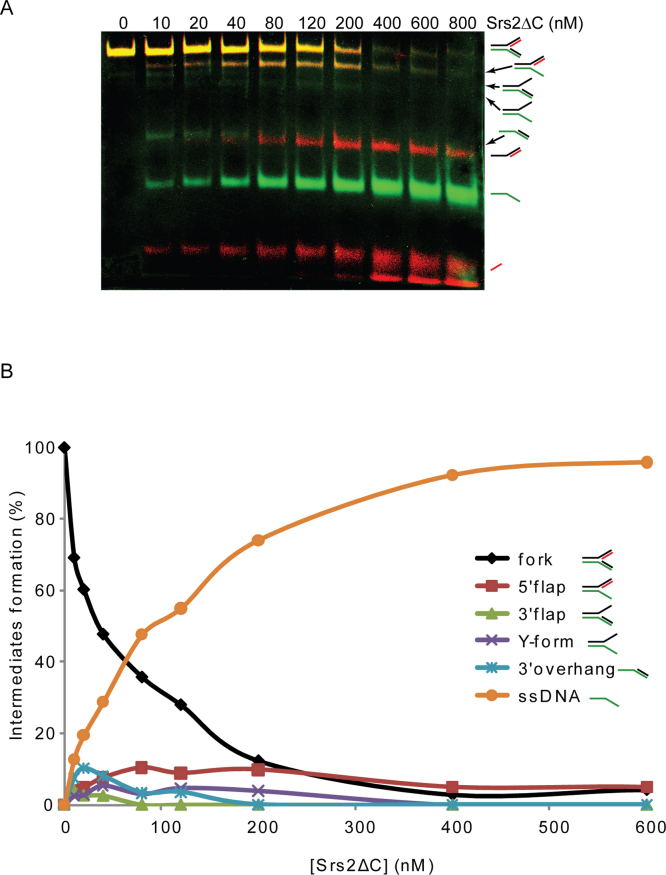
The helicase activity of Srs2ΔC was tested on a fork labelled with two fluorescent dyes, CY5 (red) and FITC (green). Increasing concentration of Srs2ΔC was incubated in buffer H with 3nM concentration of the substrate, for 10 min at 30 °C. (A) The reactions were resolved using native PAGE and analysed. (B) The plot indicates the ratio of the different DNA intermediates as function of increasing Srs2 concentration.

The above data show that Srs2 unwinds substrates mimicking both the lagging and leading forks. Together with the presence of Srs2 at replication forks, this prompted us to test the directionality of Srs2 during the unwinding of structures resembling replication forks. To address this, we used a DNA substrate containing two fluorescent dyes (3′ flap with FITC and CY5 labels at oligo1 and 4 respectively, Table 1, Fig. 3B and C). The incubation of increasing amounts of Srs2ΔC with such a substrate led to the generation of all possible intermediates with no obvious preferred initiation of unwinding (Fig. 3B). Among the products, we noticed that 3′ overhang is not accumulated but further unwound into ssDNA, reflecting the highest unwinding affinity for this substrate (Fig. 3A). We also performed a time-dependence experiment at a low Srs2ΔC concentration to identify the initial products of unwinding (Fig. 3C). In this experiment, in only 1 min, we observed the first products of flap unwinding, 3′ overhang and Y-form DNA (Fig. 3C). Both ssDNA products were observed simultaneously 2.5 min after the start of the reaction (Fig. 3C). Moreover, testing with the dual-labelled fork substrate (fork with FITC and CY5 labels at oligo1 and 3, respectively, Table 1) also showed the concomitant appearance of all of the intermediates at various protein concentrations (Supplementary Fig. 2), indicating that Srs2ΔC is able to unwind both the leading and lagging strand as well as the parental duplex without preferred directionality.

### 3.4 RPA inhibits the unwinding of Srs2

**Fig. 4:**
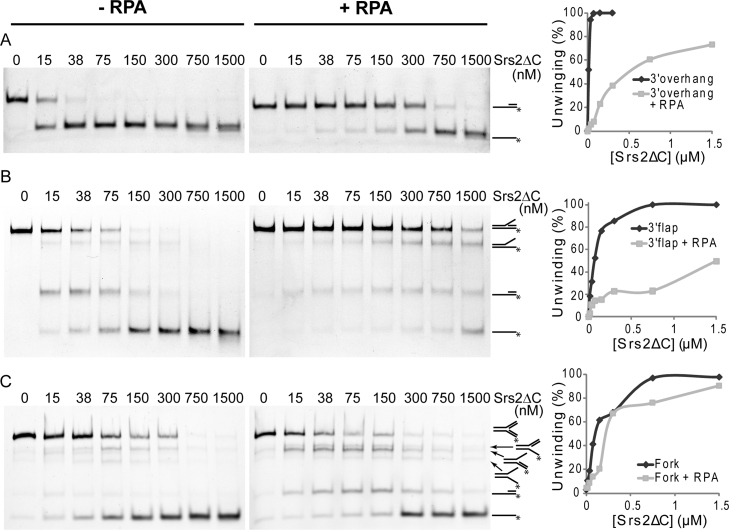
Effect of RPA on the helicase activity of Srs2ΔC. The assay for helicase activity was performed as described in the experimental procedures, except the FITC-labelled DNA substrate was preincubated for 2 min at 25 °C either in the absence (left) or presence (right) of RPA (190 nM). The DNA substrates analysed represent 3′ overhang (A), 3′ flap (B) and fork (C) structures. The plots represent the formation of ssDNA.

The ability of Srs2 to unwind an obstructing DNA strand on both the leading and lagging strand template led us to hypothesise that the presence of a single-strand DNA binding protein (such as RPA) or Srs2-interacting partners might influence the unwinding. Therefore, we performed a standard helicase assay with Srs2ΔC on several DNA substrates pre-coated with RPA. In the presence of RPA, we observed a significant inhibition of the unwinding of the 3′ overhang and 3′ flap substrates (Fig. 4A and B). Only the unwinding of the fork DNA substrate showed a minimal response to the presence of RPA, reflecting the absence of ssDNA in the structure (Fig. 4C). Comparable results were also obtained with full-length Srs2 (Supplementary Fig. 1).

**Supplementary Fig. 3:**
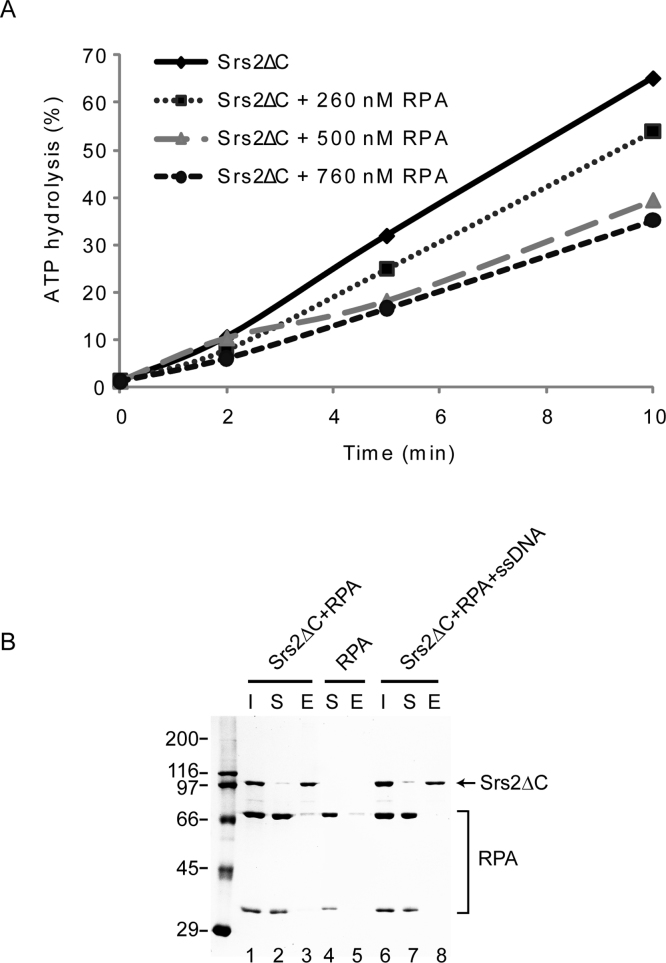
(A) Plot showing the ssDNA-dependent ATPase activity of Srs2ΔC over time, in the absence or in the presence of RPA. The reactions were prepared as described in experimental procedures. To see the effect of RPA, ssDNA (15 μM nucleotides) was preincubated with the indicated amounts of RPA before adding Srs2ΔC (100 nM). (B) Physical interaction between Srs2ΔC and RPA. RPA (6 μg) was incubated with Ni-NTA agarose coated with Srs2ΔC (6 μg), in the absence (lanes 1–3) or in the presence of ssDNA (lanes 6–8). In this case, the beads were afterwards incubated with DNase I, washed, and treated with SDS to elute bound proteins. RPA was also mixed with Ni-NTA agarose alone (lanes 4 and 5) as a control. The input (I), the supernatant that contained unbound proteins (S), and the SDS eluate (E) were analysed by SDS-PAGE.

**Supplementary Fig. 4:**
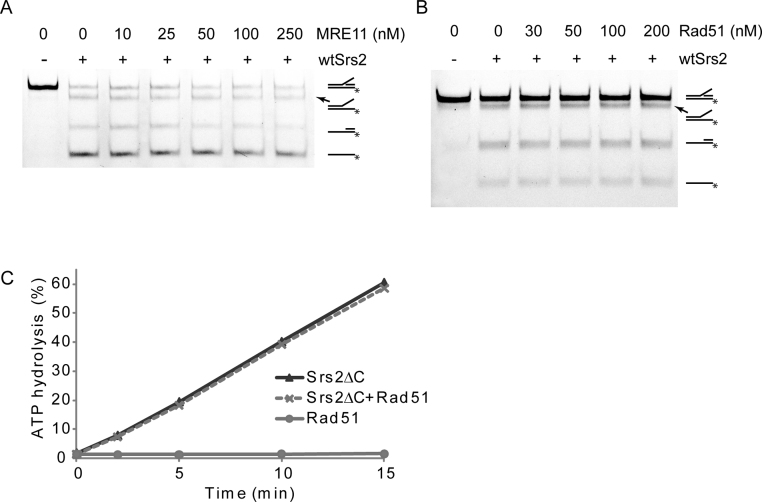
Mre11 and Rad51 do not represent an obstacle for Srs2. (A) Helicase activity on 3′ flap (3 nM) with 25 nM full-length Srs2 in the presence of increasing concentration of Mre11. (B) Helicase activity assay on 3′ flap (3 nM) of full-length Srs2 (20 nM) in the presence of the indicated amounts of Rad51. (C) ATPase activity of Srs2ΔC in the absence and presence of Rad51, performed as described in experimental procedures.

Furthermore, the inhibitory effect of RPA on Srs2 was also monitored using its ssDNA-dependent ATPase activity. The ssDNA was pre-coated with RPA followed by incubation with Srs2ΔC and radiolabelled ATP. As shown in supplementary Fig. 3A, subsaturating ratios of RPA versus ssDNA (1 RPA molecule: 2 binding sites) resulted in marked inhibition of the ATPase activity. An additional increase in the RPA concentration resulted in a 50% reduction of Srs2ΔC ATPase activity, indicating that RPA represents an obstruction to Srs2 translocation. No further inhibition was observed when an excess of RPA in relation to ssDNA was used (Supplementary Fig. 3A). To test whether this inhibition is due to direct physical interaction between Srs2ΔC and RPA, we used a pull-down assay. Purified Srs2ΔC was incubated with RPA and then immobilised on Ni-NTA beads. However, under these conditions, we were not able to detect a direct interaction between Srs2ΔC and RPA. In addition, pre-incubation of RPA with ssDNA did not result in a detectable interaction between these two proteins (Supplementary Fig. 3B). To determine whether other DNA- or Srs2-interacting proteins could also present an obstacle to or affect Srs2 activity, we tested the effect of the presence of the Rad51 and Mre11 proteins. Interestingly, neither Rad51 nor Mre11 had any effect on the ability of wtSrs2 to unwind DNA substrates or Srs2ΔC to hydrolyse ATP (Supplementary Fig. 4), indicating that only RPA binding to ssDNA is able to reduce both the helicase and the ssDNA-dependent ATPase activities of Srs2ΔC.

### 3.5 Srs2 helicase activity on recombination intermediates

**Supplementary Fig. 5:**
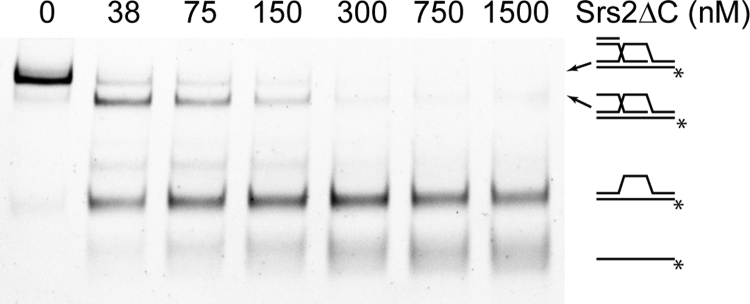
Helicase activity of indicated concentrations of Srs2ΔC on a D-loop substrate (3nM), performed as described in experimental procedures.

**Fig. 5:**
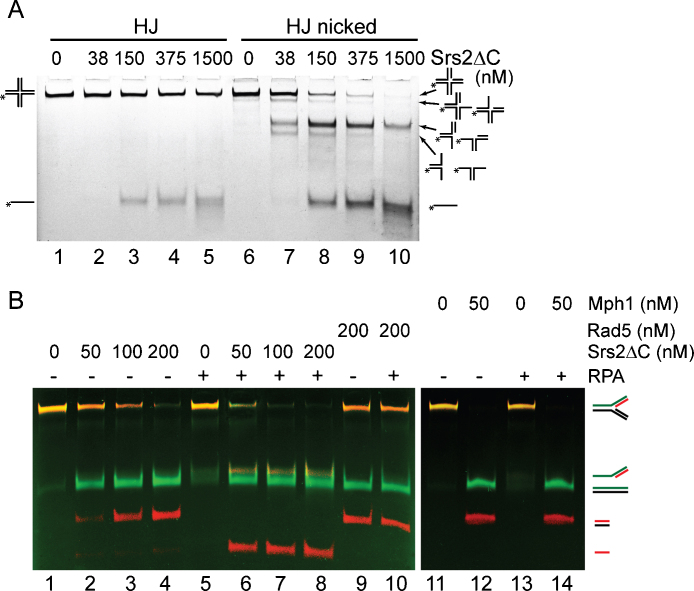
Helicase versus branch migration activity on rigid and mobile DNA structures. (A) Helicase activity of Srs2ΔC on HJ with or without a nick. Reaction mixtures containing FITC-labelled DNA (3 nM) were incubated with the indicated amount of Srs2ΔC for 10 min at 30 °C and then analysed. (B) The ability of Srs2 to branch migrate a fork substrate containing partial homology. The substrate (6 nM, labelled with CY5 (red) and FITC (green)) was incubated for 15 min at 30 °C with the indicated amounts of Srs2ΔC (lanes 1–8), Rad5 (lanes 9–10) or Mph1 (lanes 11–14). For samples in lane 5–8, 10 and 13–14, the DNA substrate was preincubated with 100 nM RPA for 5 min at 4 °C.

Genetic studies indicate that Srs2 promotes SDSA [29,43]. This prompted us to test its ability to unwind recombination intermediates, such as HJs or D-loop structures. Increasing concentrations of Srs2 were only partially able to unwind HJ-like structures (Fig. 5A, lanes 1–5). In contrast, the introduction of a nick into the HJ structure resulted in almost complete unwinding at 1.5 μM of Srs2, indicating that the presence of a discontinuity in duplex DNA significantly increases the unwinding by Srs2 (Fig. 5A, lanes 6–10). Similarly, the synthetic D-loop structure also appeared to be a very good substrate for Srs2 helicase activity, showing preferential unwinding of the short oligonucleotide on the invading strand or the invading strand itself (Supplementary Fig. 5).

**Supplementary Fig. 6:**
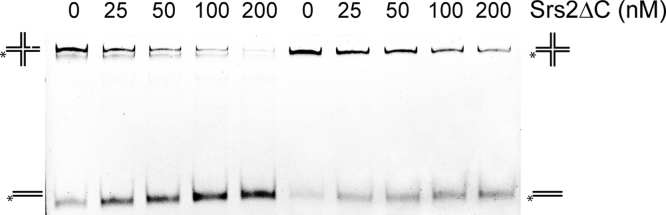
The activity of Srs2ΔC to unwind mobile HJ and nicked HJ containing partial homology labelled with FITC. The substrate (6 nM) was incubated for 15 min at 30 °C with the indicated amounts of Srs2ΔC.

Other DNA repair translocases have been shown to possess an intrinsic branch migration activity [44,45]. To test whether Srs2 has such an ability, we designed a fluorescently labelled mobile fork (Table 1) and tested the ability of Srs2 to branch migrate this substrate. Incubation of Srs2 with such DNA structures produced both longer and shorter duplexes, the two products of branch migration (Fig. 5B, lanes 1–4). Similarly, Srs2 was also able to generate the expected reaction products from mobile HJ and nHJ (Supplementary Fig. 6). However, it could also be possible that these products are the results of Srs2 helicase activity followed by the reannealing of unwound oligonucleotides. To prevent such reannealing, we performed the reaction in the presence of RPA. Under these conditions, we did not observe any short duplex DNA products; instead, 5′ overhang and short single-strand DNA were detected, indicating that these species represent the products of a helicase activity (Fig. 5B, lanes 5–8). Indeed, in the case of the Rad5 and Mph1 proteins, a previously described branch migration proteins [44,45], the presence of RPA did not have any effect on the resulting reaction products (Fig. 5B, compare lanes 9 and 10 and lanes 11–14). These data indicate that Srs2 possesses a helicase but no branch migration activity.

## 4 Discussion

Previous studies have suggested that Srs2 functions by acting at replication forks as well as at recombination intermediates formed as a result of replication stalling or DSB repair (reviewed in Marini and Krejci [7]). In this study, we wanted to further corroborate on previous studies [13,14] and characterise in details the DNA binding and helicase activities of the Srs2 protein to shed light on its multiple roles during DNA replication/recombination. Initially, we tested the ability of Srs2ΔC to bind various DNA substrates that would imitate those naturally occurring in vivo, including different fork structures, 3′ or 5′ overhangs, and single- and double-strand DNA. The highest binding preference was observed for structures containing ss/ds DNA junctions (Fig. 1). This specificity is in an agreement with the competition assays demonstrating that a structure containing a ss/dsDNA junction is bound with much higher affinity compared to ssDNA and is able efficiently outcompete ssDNA from the protein–DNA complex (Fig. 2). We conclude that Srs2ΔC has a specific preference for an ss/dsDNA junction, without a preference for structures with either a 3′ or 5′ tail. The bacterial Srs2 homologue, UvrD, shows similar affinities. However, it has a clear preference for binding substrates containing a 3′ extension [46].

An examination of whether the binding mode provides a foundation for the ability of Srs2ΔC to unwind various DNA substrates revealed similar unwinding efficiencies for the majority of substrates, including the fork, 3′ flap, 5′ flap, and Y-form. Interestingly, without exhibiting any specific binding preference, the 3′ overhang was unwound the most efficiently. The potential structural feature that causes the preferred unwinding of the 3′ overhang remains to be determined. It cannot be explained simply by the generation of competing intermediates in the case of more complicated DNA substrates, as similar substrates (5′ overhang and Y-form) show reduced unwinding. It is tempting to speculate that the interaction of Srs2ΔC with single-strand DNA at the 3′ end of DNA could directly promote unwinding in the 3′ to 5′ direction [13]. However, a similar unwinding preference was not observed for the 3′ flap. However, despite the comparable affinity of Srs2ΔC towards dsDNA, it unwinds the blunt duplex DNA poorly (Fig. 3). This may be related to the fact that the ability of Srs2 to initiate unwinding from discontinuities within the DNA was also observed for its bacterial homologue UvrD [47,48].

Srs2 was shown to associate with replication forks in vivo [24,33,34], and our data demonstrate that it can directly target and efficiently unwind various fork substrates. Therefore, we tested the preferred polarity of fork unwinding dictated by the presence of a leading or lagging strand to help understand its biological role during DNA replication. In experiments using dual labelled substrates, we did not observe any single primary product, but rather found the simultaneous generation of the individual products that are the result of the unwinding of the 3′ flap as well as fork substrates (Fig. 3B and Supplementary Fig. 2). Our time-course experiment revealed the same unwinding rates for the individual intermediates (Fig. 3C). Srs2ΔC thus unwinds both leading and lagging strands as well as the parental duplex without any obvious preference, suggesting that it could act on diverse stalled replication forks to promote appropriate repair. This could help to explain the ability of Srs2 to unwind various hairpin-forming structures and the observed role of Srs2 in preventing fragility and instability in CAG/CTG repeats [38,41,49]. Furthermore, the helicase activity of Srs2 was shown to be necessary for replication to proceed past the hairpin [38].

Srs2ΔC appears to bind fork structures at the branch point and translocate in all three directions with no preference while keeping to the 3′ to 5′ direction of translocation. This is different from other homologous helicases, including Rep, PriA and UvrD, which show preferred unwinding of the lagging strand DNA within the 3′ flap structure [47,50]. The specificity could be dictated by another DNA-binding domain outside the truncated version of Srs2 used in this study. However, we did not observe any difference in unwinding using full-length Srs2 (Supplementary Fig. 1).

**Supplementary Fig. 7:**
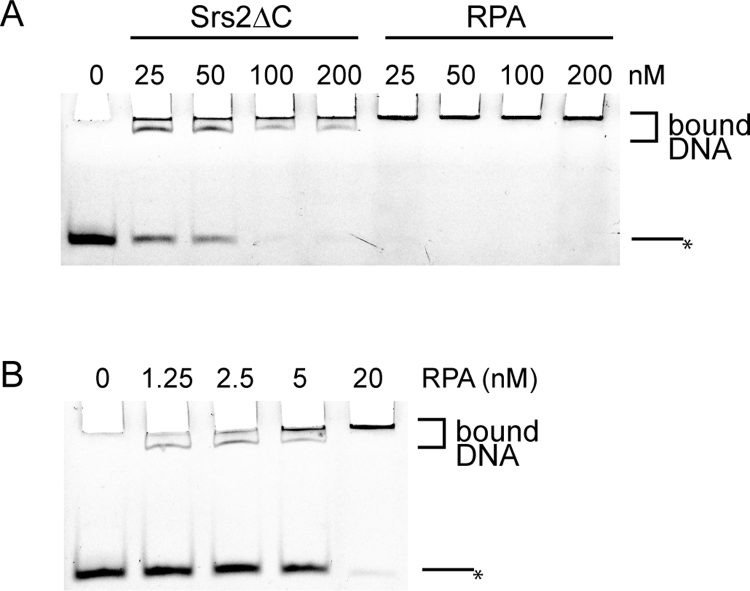
RPA has higher affinity to ssDNA than Srs2ΔC. (A) Reaction mixtures containing FITC-labelled ssDNA (oligo1, 3 nM) were incubated together with the same amounts of Srs2ΔC or RPA for 10 min at 30 °C and analysed by EMSA. (B) The same reaction as in A except lower amounts of RPA was used for EMSA.

The ability of Srs2 to dissociate fork structures in all three directions indicates that its initial orientation of binding is not determined by an asymmetry of the junction. Thus additional factors might be needed in vivo to ensure that Srs2 binds in a way that is productive for fork unwinding. Therefore, we tested an effect of the single-strand binding protein RPA on Srs2 helicase activity. While the presence of a lagging or leading strand did not have a significant effect on unwinding, coating ssDNA regions with RPA resulted in a suppression of Srs2 helicase activity (Fig. 4 and Supplementary Fig. 1). Correspondingly, ATPase activity was also inhibited by the presence of saturating amounts of RPA. This is in contrast to previously published data in which RPA was able to stimulate the unwinding of long DNA substrates by Srs2 [13]. However, in the case of large DNA substrates, this could reflect a role of RPA to sequester unwound ssDNA, prevent re-annealing and/or suppress the formation of secondary structures of DNA. Our data show that RPA presents an obstacle for the translocation of Srs2 along ssDNA, indicating that it could inhibit unscheduled unwinding of the replication fork in vivo. This could be explained by the higher affinity of RPA towards ssDNA (Supplementary Fig. 7). In addition, Srs2 cannot alleviate this inhibition via a direct interaction with RPA, as we were not able to detect it under our experimental conditions. Even the pre-incubation of RPA with ssDNA, which was shown to be necessary for the interaction between RPA and Rad52 [51], did not result in any detectable interaction between Srs2ΔC and RPA (Supplementary Fig. 3B). In contrast, testing Srs2-interacting proteins, including Rad51 and Mre11, did not show any inhibitory effect on Srs2 helicase and ATPase activities (Supplementary Fig. 4). However, in previous work, Rad51 was shown to stimulate the Srs2-mediated unwinding of longer DNA substrates [14], which might again correspond with the ability of the Rad51 filament to either remove secondary structures or sequester unwound ssDNA. This explanation is also in agreement with the ability of Srs2 to activate the ATPase activity of Rad51 followed by the destabilisation of Rad51 association with DNA [26] and is not accompanied by increased Srs2 ATPase activity (Supplementary Fig. 4). Our data would suggest that, due to the direct protein interaction between Srs2 and Rad51 or Mre11, both proteins do not present an obstacle for Srs2 translocation and could even serve for Srs2 targeting to the recombination intermediates.

Our data point to the ability of Srs2 to act directly on replication forks. Indeed, a recent study indicated that overexpression of Srs2 primarily affects the progression of DNA replication, as it causes the activation of the replication checkpoint [37]. Furthermore, the deletion of many key replication components leads to synthetic lethality when Srs2 is overexpressed. The helicase-dead mutants induce a delay in S-phase progression and show enhanced lethality in combination with genes involved in DNA repair, suggesting that the helicase activity impedes the proper processing of the appropriate DNA repair pathway [37].

Srs2 has been shown not only to block inappropriate recombination [22,23] but also to promote the SDSA pathway of HR [29]. Therefore, we tested the ability of Srs2 to act on various recombination intermediates, including HJs and D-loops. As observed previously [14], Srs2 is unable to unwind efficiently a HJ. Interestingly, the introduction of a nick resulted in a dramatic stimulation of the HJ unwinding. This is in contrast to a previous study by Dupaigne et al. [14] and could not be simply explained by the requirement for an ssDNA region as we also observed high efficiency in unwinding the fork structure. This rather argues for structural features of branched DNA substrates or presence of ss/dsDNA junction to initiate the disruption. This is further supported by the observation that the introduction of a nick into duplex DNA also resulted in the dramatic stimulation of the unwinding activity compared to dsDNA (Fig. 3A). In agreement, similar initiation of unwinding was observed for UvrD homologue [47,48]. Next, we directly assessed whether Srs2ΔC can branch migrate HJ or catalyse fork regression. However, in contrast to other branch migrating enzymes (such as Rad5 and Mph1), Srs2ΔC does not process recombination/replication intermediates through branch migration or fork regression but rather solely through its helicase activity.

In summary, our data help us to understand the possible role of Srs2 in processing stalled or blocked replication forks as part of postreplication repair as well as HR. Interestingly, while Srs2 does not have a direct mammalian homologue, several human helicases have preserved its anti-recombinase function, including RECQL5, BLM and FANCJ [52–54]. Furthermore, studies of the human FBH1 helicase have shown that it can regulate the recombination instead of Srs2 in yeast [55], suggesting that it can substitute for most of the Srs2 activities. Just recently another protein, called PARI, was suggested to be a mammalian Srs2 functional homologue that suppresses inappropriate recombination at replication forks [56]. It will also be interesting to see how other interacting proteins influence Srs2 to promote their activity at the replication fork or in the SDSA pathway of homologous recombination.

## Conflict of interest

The authors declare that there are no conflicts of interest.

## Figures and Tables

**Table 1:**
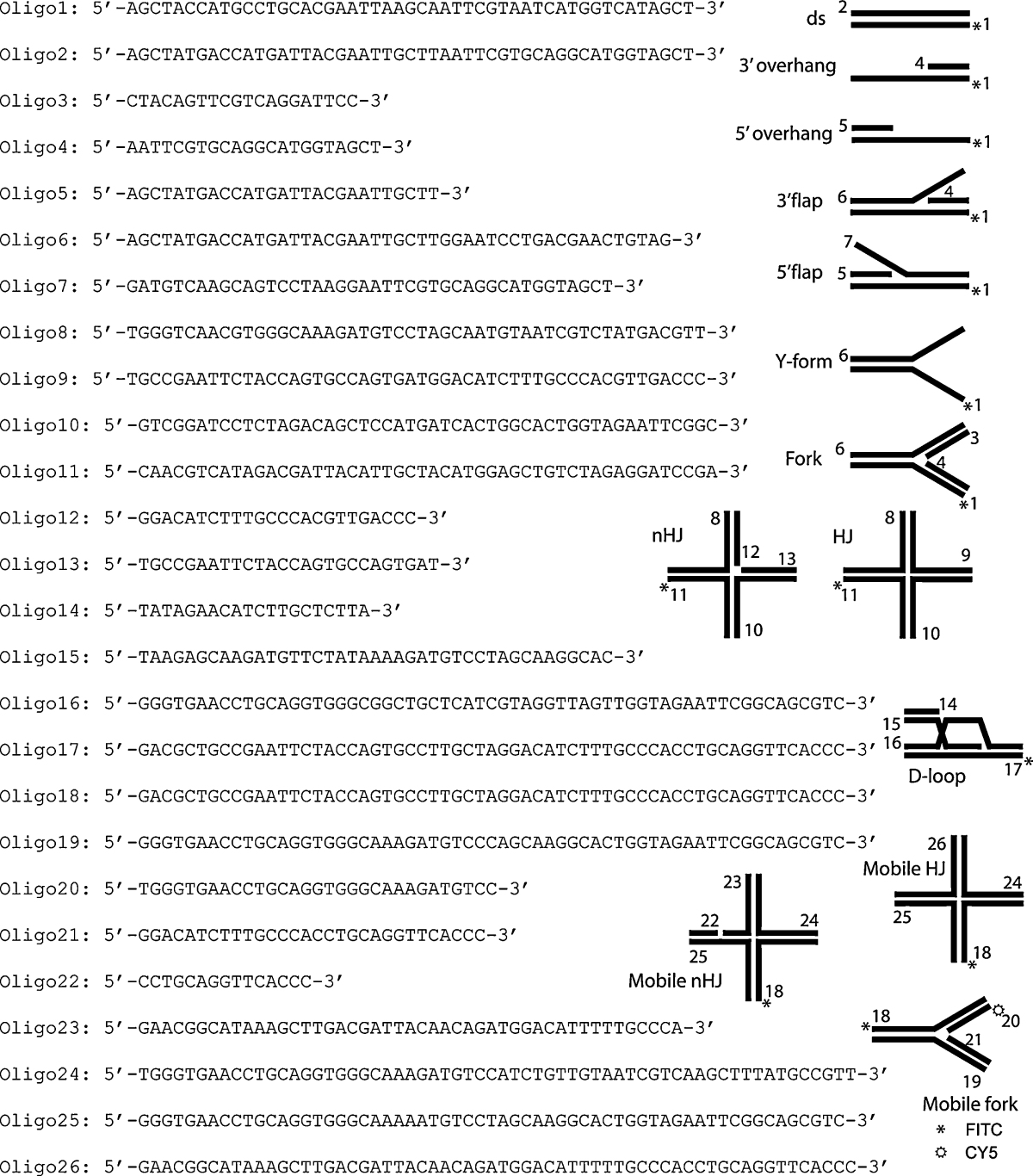
DNA substrates used in this study.

The list of sequences of oligonucleotides used to make individual synthetic substrates. Each DNA substrate is made from the oligonucleotides indicated by the number on each representative schematic. The number is positioned at the 5′ end of its respective oligonucleotide. The asterisks denote end modification by a fluorescent dye (fluorescein or Cy5).
